# Ridge splitting using autogenous bone wedge versus the conventional intercortical augmentation technique in horizontally deficient anterior maxilla: a randomized clinical trial

**DOI:** 10.1186/s12903-025-06345-z

**Published:** 2025-06-21

**Authors:** Mohammed Omara, Sameh Mekhemer, Sally Mansour, Yasmin Ahmed

**Affiliations:** 1 Oral and Maxillofacial Surgery Department, Faculty of Dentistry, Cairo University, 11 Saraya Street, Manial, Cairo Egypt; 2 Oral and Maxillofacial Radiology Department, Cairo University, Cairo, Egypt

**Keywords:** Ridge splitting, Bone wedge, Mixed xenograft and autogenous bone particles, Horizontally deficient ridge, Aesthetic zone, Ridge augmentation

## Abstract

**Objectives:**

This study aims to evaluate the quality and quantity of gained and maintained bone width after Ridge splitting utilizing autogenic bone wedge versus mixed bone particles for horizontal ridge augmentation in the anterior aesthetic zone.

**Materials and methods:**

This randomized clinical trial included 20 patients with horizontally deficient anterior maxillary alveolar ridges. Patients were divided equally into two groups. Group I received an autogenous bone wedge harvested from the chin area to be placed intercotically after ridge splitting (intervention group). Group II received mixed bone particles of autogenic and xenogeneic bone placed intercortically after ridge splitting (control group). Radiographic assessment of gained and maintained alveolar bone width at three vertical levels was performed using CBCT at three-time intervals (preoperative, immediate postoperative, and 6 months postoperative). Histologic and histomorphometric analysis of core biopsy harvested immediately before implant placement was also performed to assess bone quality and % of newly formed bone area using H&E and Mansons trichrome stains. Collected data were conducted for statistical analysis.

**Results:**

The outcome of the studied grafts showed a significant increase of the immediate postoperative bone width in the control group more than the intervention group, with a mean difference from the preoperative bone width (2.17 ± 1.10) mm for the control group and only (1.44 ± 0.66) for the intervention group. In contrast, the 6-month postoperative bone width was decreased in both groups with a mean difference from the immediate postoperative bone width (1.21 ± 0.54) in the control group (p-value < 0.001) compared to only (0.41 ± 0.50) in the intervention group (p value = 0.135); this statistical data revealed that the bone wedge technique of the intervention group helped to maintain the gained bone width more than the packed bone particles of the control group. Moreover, the intervention group showed higher value and quality of newly formed mature bone with well-formed havarsian canals than the control group, which showed lower bone quality of osteoid and fibrous tissue with remnants of xenogenic bone particles microscopically.

**Conclusions:**

The two-stage ridge-splitting procedure using an interposition bone wedge is an effective method for horizontal ridge augmentation in the horizontally deficient anterior maxilla.

**Clinical relevance:**

The two-stage Ridge splitting with an inter-positional bone wedge ensures better bone width maintenance and quality.

**Trial registration:**

The study was registered on ClinicalTrials.gov on 24/07/2024 under the registry number “NCT06529653”. It adhered to the Declaration of Helsinki on medical research ethics and received approval from the Institutional Research Ethics Committee of the Faculty of Dentistry, Cairo University (IRB number: 161022).

**Supplementary Information:**

The online version contains supplementary material available at 10.1186/s12903-025-06345-z.

## Introduction

Deficient bone width of the anterior maxilla is a significant challenge requiring proper management before implant placement. Adequate bone width is essential for the correct positioning and orientation of root-form implants [1]. Cawood and Howell proposed a widely accepted classification system for alveolar ridge resorption, describing six classes of bone loss. Class IV is a common feature of the resorbed anterior maxillary Ridge after long-standing anterior tooth loss. This pattern of bone loss often fails to provide sufficient width to accommodate the diameter of endosseous implants or allow placement in a prosthetic-driven position [2].

Various bone grafts and augmentation techniques have been employed to address these deficiencies. Bone grafting materials include autogenic, allogeneic, xenogeneic, synthetic bone, and combinations [3]. Among these options, autogenic bone grafts remain the gold standard due to their osteoconductive and osteoinductive properties. Numerous surgical techniques have been described to manage horizontal bone defects in the anterior maxilla, ranging from alveolar ridge plateauing and bone expansion to ridge splitting, onlay bone grafting, titanium mesh application, and guided bone regeneration. Each of these techniques has specific advantages and limitations, making it essential to select the most appropriate method based on the radiographic features and architecture of the alveolar Ridge [4, 5].

Ridge splitting is a well-established surgical approach in implant dentistry for overcoming horizontal ridge deficiencies. This technique can be categorized based on timing: non-staged, where implant placement is performed simultaneously, or staged, where implant placement is delayed and performed 4–5 months after horizontal ridge grafting. Variations in the ridge-splitting technique include variations in flap design, whether full-thickness or partial-thickness [6, 7]. Additionally, the type of grafting material, including autogenous, xenogeneic, or allogeneic bone, and the form of the bone graft, whether blocks or particles are important considerations. The timing of implant placement, whether immediate or delayed, also significantly influences clinical outcomes [8].

For better outcomes, staged Ridge splitting with interposition graft materials has been introduced to provide adequate ridge dimensions for prosthetic-driven delayed implant placement. The containment of these materials sandwiched between the split cortical shells validates the interposition bone grafting over the onlay bone placement as it enhances the graft-host interaction. Cortellini et al. demonstrated that bone fill is improved when the number of residual defect walls increases [9]. Inter-positional grafting converts single-wall contact into four-wall contact, enhancing graft vascularity and promoting faster healing with minimal graft resorption. The graft placement between two pedicle bone layers accelerates angiogenesis, increasing the osteoinductive and osteoconductive potential of the graft and resulting in greater biological activity [10, 11]. Furthermore, compared to interposition bone particles, interposition block grafts are protected from detrimental forces on the alveolar Ridge, minimizing postoperative mechanical collapse [12].

So, this study aims to evaluate both the quality and quantity of gained and maintained bone width after staged Ridge splitting utilizing autogenic bone wedge versus mixed autogenic and xenogeneic bone particles as a grafting material between split cortices in the horizontally deficient anterior maxilla.

Patients and methods.

## Patients and methods

### Design and population

This randomized parallel clinical study was registered on ClinicalTrials.gov under the registry number NCT06529653. It was conducted on 20 patients complaining of edentulous maxillary aesthetic region seeking fixed prosthetic restorations. The patients were referred to the outpatient clinic of the Oral and Maxillofacial Surgery Department, Faculty of Dentistry, Cairo University. The selected patients signed informed consent forms after the treatment plan, possible complications, and side effects were explained to them.

The study was registered on ClinicalTrials.gov on 24/07/2024 under the registry number “NCT06529653”. It adhered to the Declaration of Helsinki on medical research ethics and received approval from the Institutional Research Ethics Committee of the Faculty of Dentistry, Cairo University (IRB number: 161022).

### Patient selection

Selected patients were recruited in the study according to Cone beam computed tomography (CBCT) that was performed with on-demand software (Scanora 3D-Cairo-Egypt)-360 dental scan with exposure parameters of 85 kV, 15 mA, and 6 cm field of view (FOV). Based on the following inclusion criteria: Class IV Cawood and Howel patients with long-standing horizontally deficient anterior maxillary alveolar Ridge [2] Alveolar ridges of 10 mm vertical height and 3–5 mm horizontal width., in addition to alveolar ridges with radiographically apparent intercortical spongiosa and straight cortical architecture. In contrast, patients with scoped-out labial defects or ridges with recent extractions were excluded from the study.

### Randomization and grouping

**Fig. 1:**
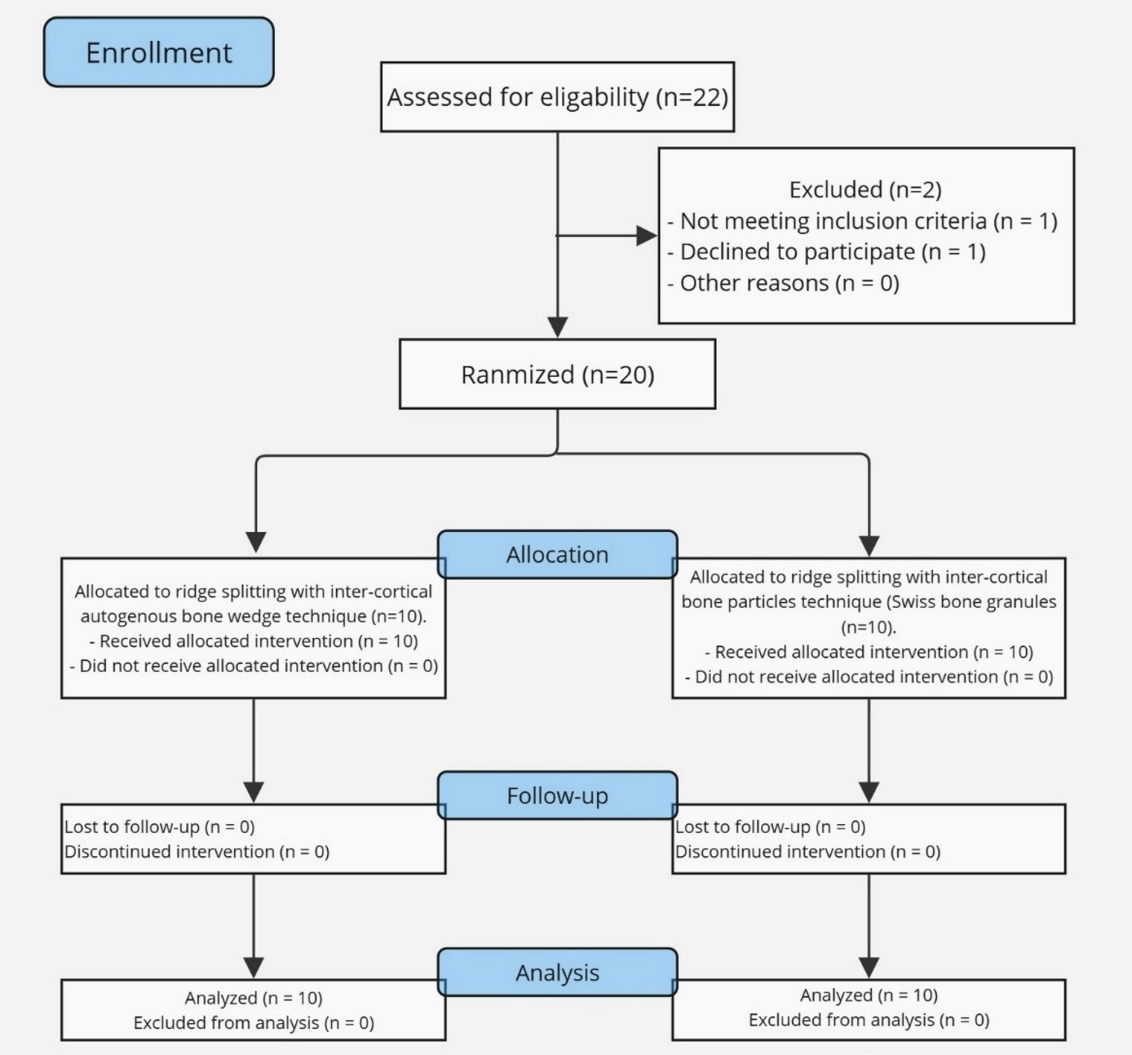
CONSORT flow chart for the studied cases

Patients were allocated randomly into two equal groups using block randomization-sequence generation (Fig. 1). The randomization was performed using two equal blocks. Twenty cards were given randomly generated sequence numbers, one number for each card. Then, these cards were placed within opaque sealed envelopes. These envelopes were placed in a container, and each participant grasped one envelope blindly on the operation’s day. The study blinding included the patients, histopathologist, radiologist, and statistician.

In the conventional group, the procedure involved Ridge splitting with inter-cortical bone particles technique through the utilization of (40% autogenic bone particles derived from mandibular symphysis mixed with 60% xenogeneic bone particles (Swiss bone 0.25 Particle size, rigid granules, made in Switzerland) followed by 6 months postoperative core biopsy and implant placement. In the intervention group, the procedure involved Ridge splitting with inter-cortical autogenous bone wedge placement derived from mandibular symphysis followed by 6 months of postoperative core biopsy and implant placement.

## Methods

### Surgical protocol

**Fig. 2:**
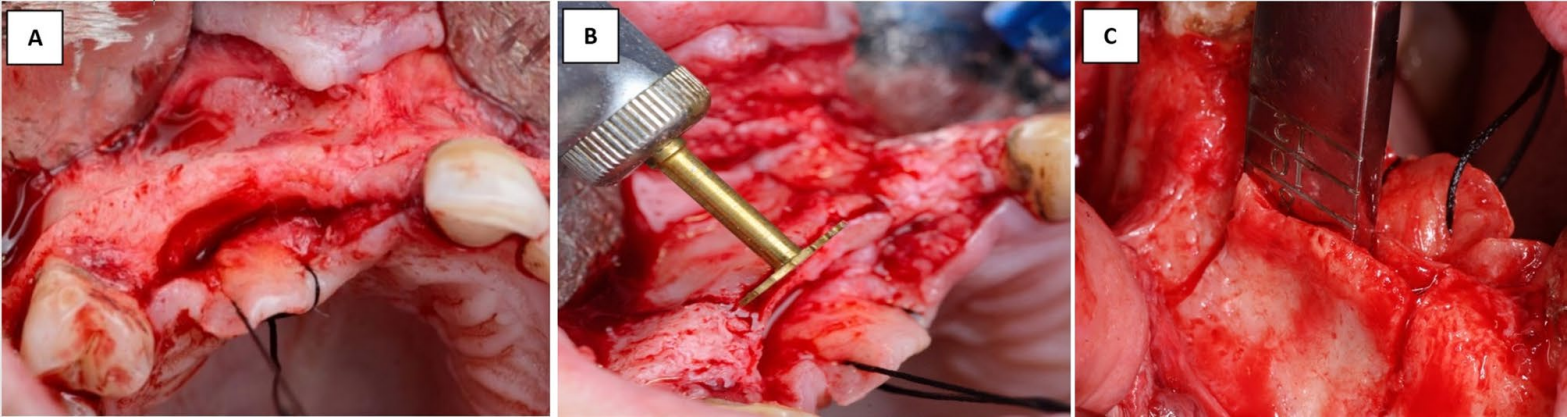
Clinical photographs illustrating steps of the ridge splitting procedure: (**A**) pyramidal flap reflection exposing the horizontally deficient Ridge, (**B**) use of a surgical disc for horizontal and vertical osteotomies, and (**C**) application of a chisel for Ridge splitting to the maximum length

Preoperative assessment included taking a history, conducting a clinical examination, and conducting a radiographic investigation. All patients underwent surgeries in the Faculty of Dentistry, Cairo University clinics. The surgical intervention was performed under local anesthesia, after betadine scrubbing and local anesthesia with 4% articaine containing 1:100,000 epinephrine. For the recipient site, a conventional full-thickness pyramidal mucoperiosteal flap was reflected to expose the facial cortical plate and ridge crest, with palatal reflection for enhanced access and tissue protection. A mid-crestal bony cut was performed using a tungsten carbide disc, followed by two vertical cuts, and the Ridge was gradually split using osteotomes tapped with a mallet to create a trough. For the donor site, a vestibular genioplasty incision was made, and a full-thickness mucoperiosteal flap was reflected to expose the anterior mandible symphysis for graft harvesting (Fig. 2).

**Fig. 3:**
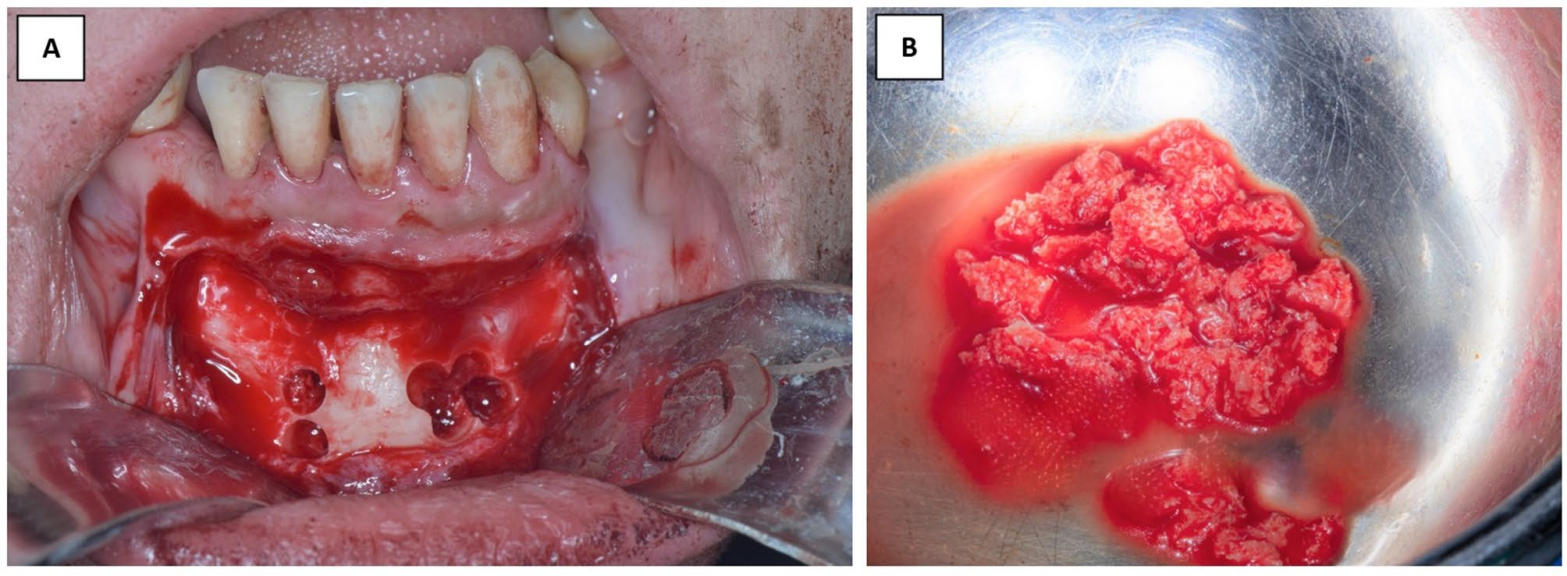
(**A**) Clinical photographs showing the donor site after harvesting bone particles using the ACM. (**B**) clinical photograph showing the mixed graft material comprising 40% autogenous bone particles and 60% xenogeneic bone particles

**Fig. 4:**
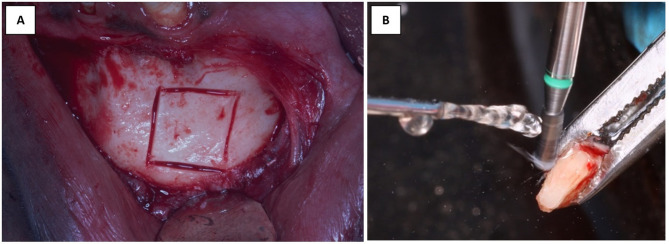
(**A**) Clinical photographs illustrating the outlined bone wedge in the chin. (**B**) Clinical photograph represents the adjustment and smoothening of the harvested wedge for smooth interposition malleting between the split cortices

In the control group, bone harvesting was performed utilizing ACM (Auto chip maker) bur and mixed with xenogeneic bone particles with (a 40–60%) ratio (Fig. 3). While in the intervention group, a surgical disc in a straight hand-piece was utilized to outline the required bone block. The superior horizontal osteotomy of the bone block should be 5 mm apical to the root apices of the mandibular anterior teeth, and the lateral borders of the bone blocks should be away from the mental neurovascular foramina. The inferior horizontal osteotomy of the bone block must be 5 mm away from the inferior border. The harvesting depth was 3–4 mm to provide a proper coronal thickness of the harvested bone wedge. To allow for proper contouring, the graft outline has to be nearly 2 mm larger than the required size in both horizontal and vertical dimensions. After bone block outlining, the bone block was totally harvested utilizing bone spreaders and straight and curved osteotomes. The harvested bone block was tailored and shaped into a bone wedge utilizing an egg-shaped bur (Fig. 4).

### Bone augmentation in the prepared recipient site

**Fig. 5:**
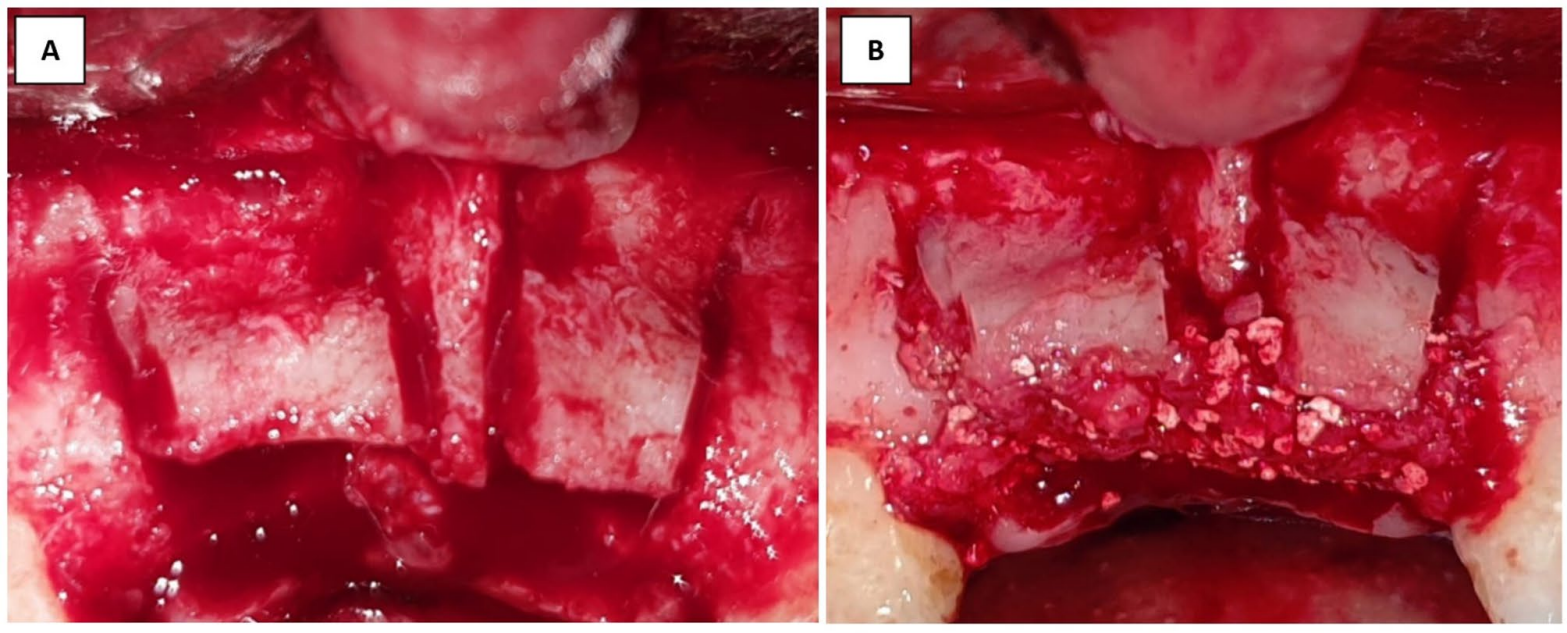
(**A**) Clinical photographs showing the horizontal and vertical osteotomies (**B**) Clinical photograph showing the split cortices after being overfilled with mixed autogenous and xenogenic bone particles

**Fig. 6:**
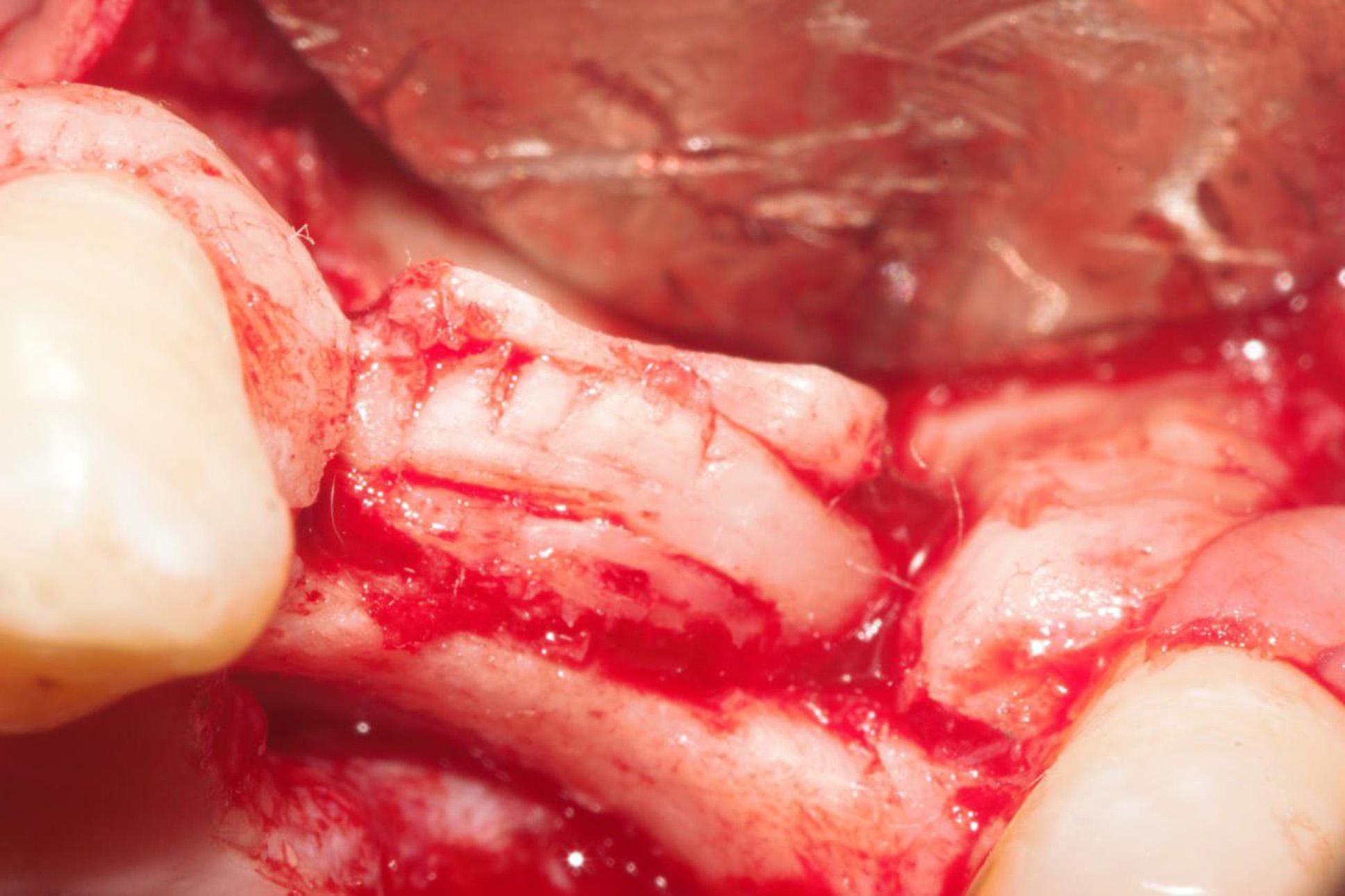
Clinical photograph for the harvested bone block (black arrow) after being wedged inter-cortically

In the control group, the mixed bone particles were packed and overfilled inter-cortically (Fig. 5). While in the intervention group, the tailored autogenic bone wedge was placed inter-cortically to the maximum length till became snugly fitted between the split cortices and diverged the split cortices to the desired bone width (Fig. 6). Suturing of both donor and recipient sites in both groups were performed using 3 − 0 vicryl suturing material (Ethicon, Norderstedt, Germany) in both groups.

### Postoperative instructions

After the closure of the wound, a pressure band was applied to the chin and lip areas for 48 h postoperatively. The patients were then instructed to apply ice packs over the chin and lip area for 20 min every hour for 6 h postoperatively and to rinse their mouth with warm saline solution starting the second day after surgery, three times per day during the first week postoperative. The patients were kept on a soft diet for the first 48 h. Postoperative antibiotics include amoxicillin 500 ( Amoxil, Epico, Egypt), analgesics, anti-inflammatory drugs Brufen 400 (Brufen, Abbott, Egypt), and mouthwash chlorohexidine.12% (Hexitol GSK, Egypt) were prescribed for 5–7 days. The postoperative follow-up to evaluate wound healing at both the donor and recipient sites was carried out every day for the first week and then every month for 6 months. Also, all patients were checked for the presence or absence of pain, numbness, swelling, infection, hematoma, and bleeding at both the donor and recipient sites.

### Core biopsy harvesting and implant placement

**Fig. 7:**
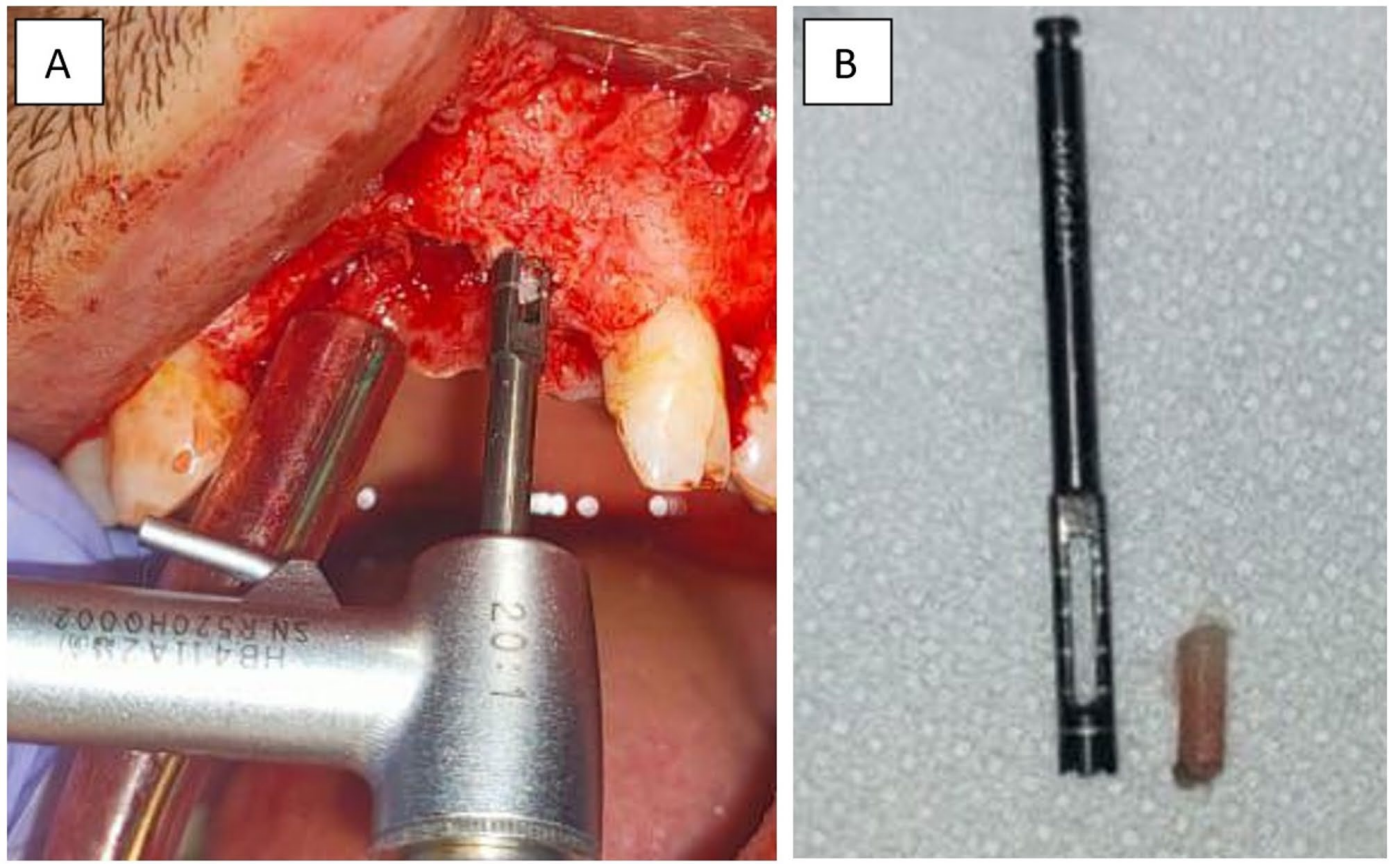
(**A**) Clinical photograph for core biopsy harvesting, (**B**) Clinical photograph showing the bone biopsy and trephine bur used for harvesting

Six months postoperatively, all augmented ridges were surgically exposed. A core biopsy using the smallest 2 mm trephine bur was harvested to be examined later for histological and histomorphometric analysis (Fig. 7). Then, implant sites were prepared, and implants were installed at the same visit. Nine months postoperatively, the patient received the final restoration.

### Histologic and histomorphometric analysis

**Fig. 8:**
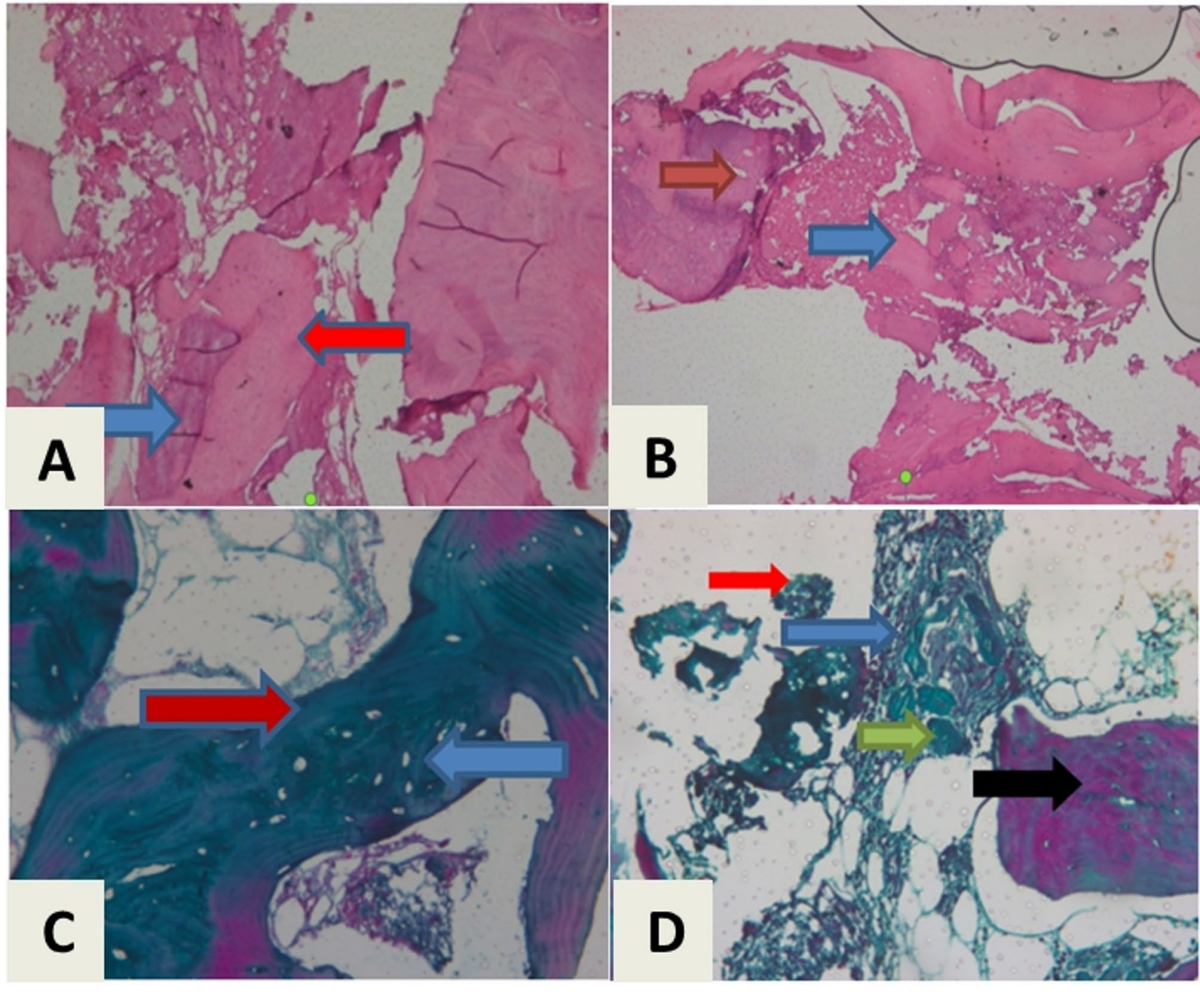
Microscopic pictures with magnification power 200 for stained specimens were harvested after 6 months postoperative at the time of implant placement. (**A**) Microscopic picture of a stained specimen from intervention group with H&E showing newly formed bone (blue arrow), original old bone (red arrow), (**B**) Microscopic picture of a stained specimen from the control group with H&E showing arrow the newly formed bone (blue arrow), original bone (red arrow), (**C**) Microscopic picture of a stained specimen from intervention group stained with Masons trichrome showing mature newly formed bone (red arrow) and lacunae of osteocytes (blue arrow), (**D**) Microscopic picture for a stained specimen from the control group with Masons trichrome showing the osteocytes (green arrow), fibrous tissue (blue arrow) and original bone (black arrow), remnants of xenograft particles (red arrow)

Histological and Histomorphometric analysis was performed using stained sections with Mayer’s hematoxylin and eosin stain (H&E) and Mason’s trichrome. The sections were examined under an Olympus CX20 microscope, with representative fields captured at 200x magnification. Images were analyzed using Image software (v. 1.45e, NIH), and bone area fractions of native and newly formed bone were calculated (Fig. 8).

### Radiographic analysis

**Fig. 9:**
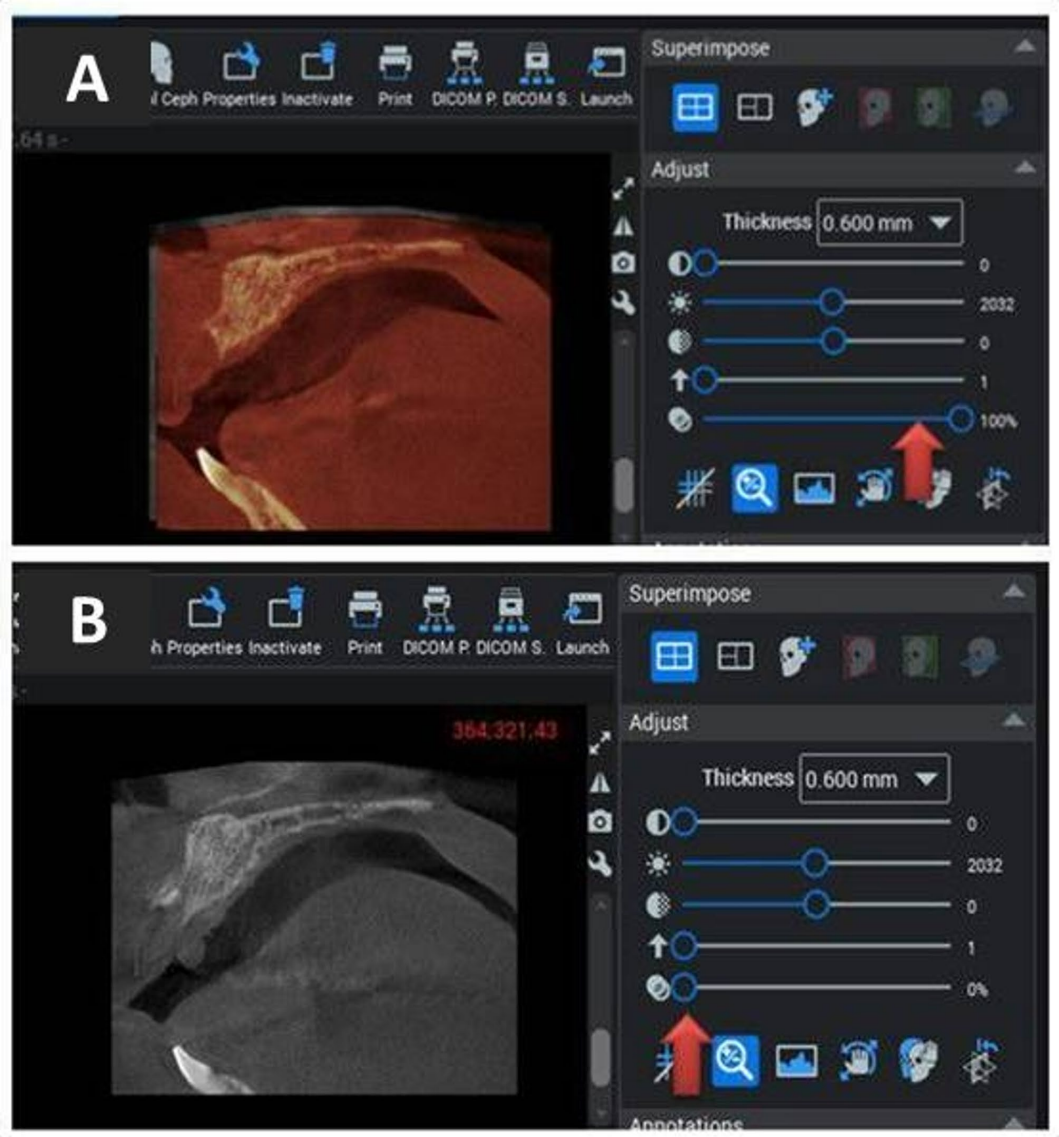
Overlay transparency tool (red arrow) adjusted to visualize the ridge scans: (**A**) preoperative scanned Ridge with the tool shifted to the right**(red arrow)**, and (**B**) immediate postoperative scanned Ridge with the tool shifted to the left. **(red arrow)**

**Fig. 10:**
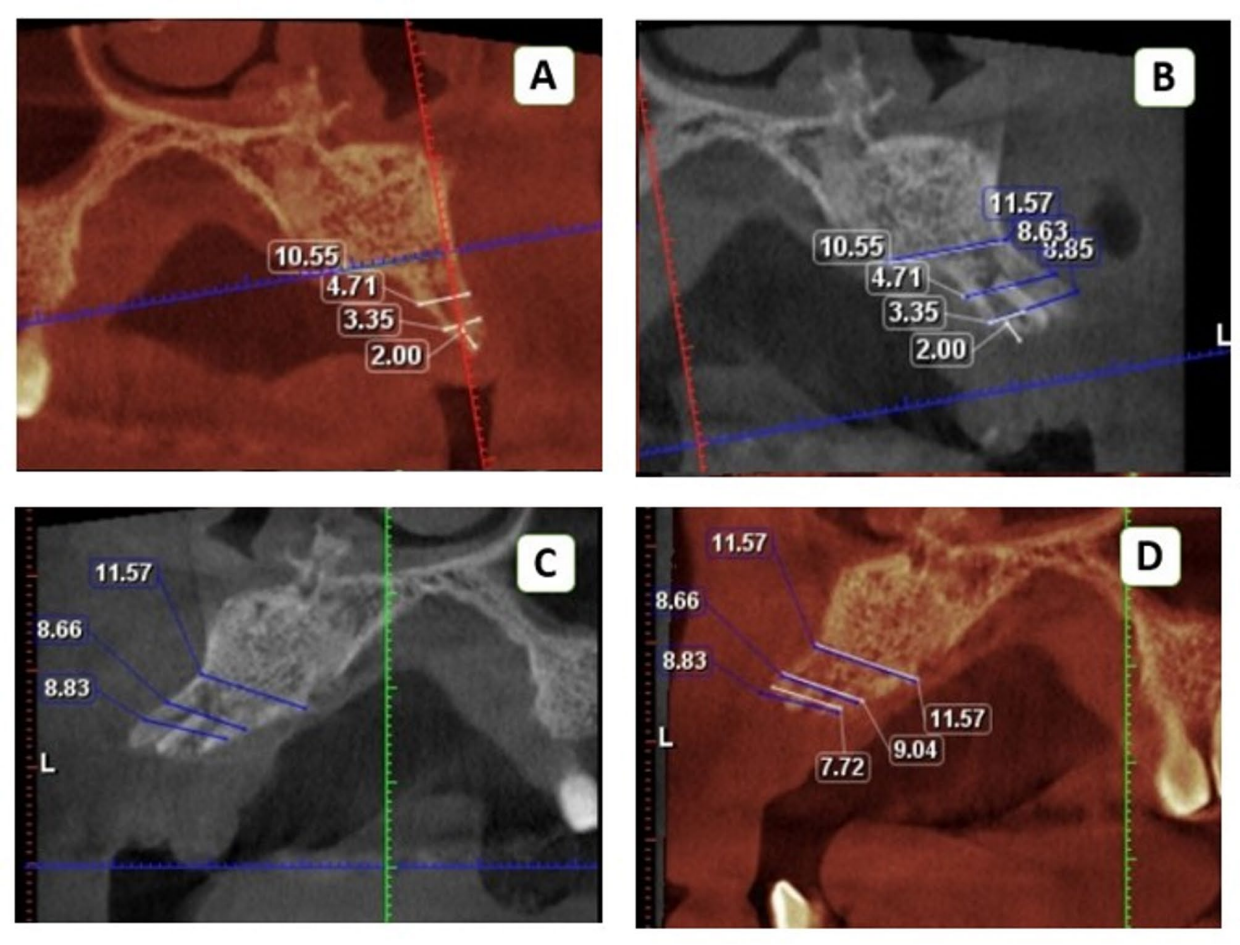
Sagittal cuts of preoperative, immediate postoperative, and 6-month follow-up superimposition scans showing bone width measurements at three vertical levels in the intervention group (**A**) Preoperative scan with white measurements for the right side of the Ridge where the implant would be placed (red shadow window). (**B**) Superimposed immediate postoperative scan with blue measurements- for the same side of the Ridge- over the preoperative scan with white measurements where the implant would be placed (white shadow window). (**C**) Immediate postoperative scan with blue measurements for the right side of the Ridge where implants would be placed (white shadow window). (**D**) Superimposed 6-month follow-up scan with white measurements for the same side over the immediate postoperative scan (blue measurements) (red shadow window)

**Fig. 11:**
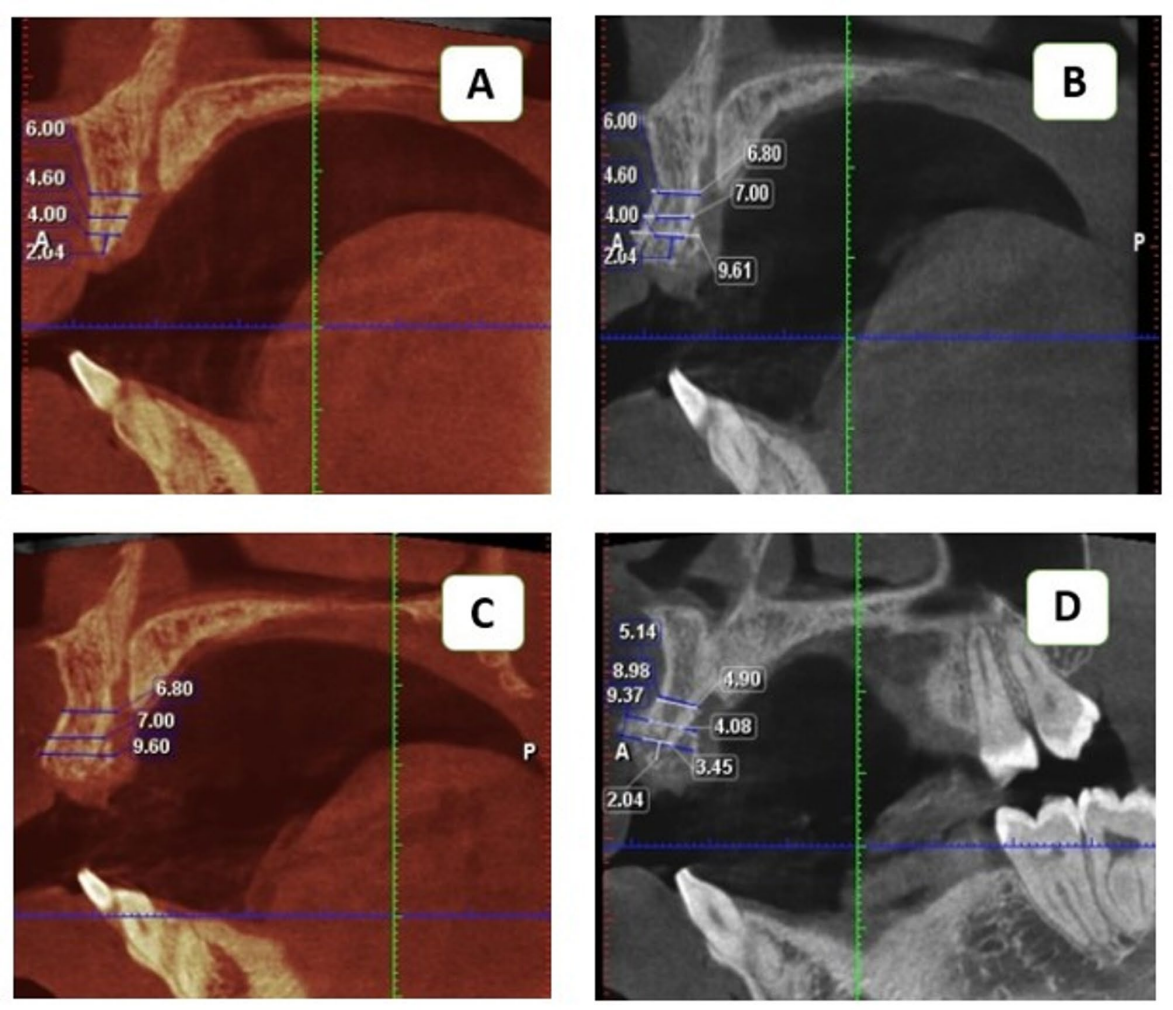
Sagittal cuts of preoperative, immediate postoperative, and 6-month follow-up superimposition scans in the conventional group, showing bone width measurements at three vertical levels. (**A**) Preoperative scan with blue measurements for the right side of the Ridge where the implant would be placed (red shadow window). (**B**) A superimposed immediate postoperative scan with white measurements over the preoperative scan for the same side of the Ridge where the implant would be placed (white shadow window). (**C**) An Immediate postoperative scan with blue measurements for the right side of the Ridge where the implant would be placed (red shadow window). (**D**) A superimposed Six-month follow-up scan with white measurements over immediate postoperative scans with blue measurements for the same side where the implant would be placed (white shadow window)

Preoperative, immediate, and 6-month postoperative CBCT scans were analyzed using Planmeca Romexis 6 software. Standardized measurements were compared via the superimposition module with a three-point fitting tool, utilizing adjustable overlay transparency to differentiate preoperative (red shadow) and postoperative (white shadow) scans (Fig. 9). The amount of gained and maintained bone during the three phases was compared (Figs. 10 and 11).

### Statistical methods

Categorical data were represented as frequency (n) and percentage (%) and analyzed using the Chi-Square test. Numerical data were explored for normality by checking the data distribution, calculating the mean and median values, and using Kolmogorov-Smirnov and Shapiro-Wilk tests. If the data was found to be normally distributed, it was presented as mean, and standard deviation values and an Independent t-test was used for the analysis. If the assumption of normality was found to be violated, the data was presented as median and range values and analyzed using the Mann-Whitney U test. The significance level was set at *p* ≤ 0.05 for all tests. Statistical analysis was performed with IBM.^®^ SPSS^®^ Statistics Version 26 for Windows.

## Results

A total of 20 patients (10 males and 10 females) with an average age of 31 years (range of 20–43 years) with horizontally deficient edentulous ridges were enrolled in the study. The deficient ridges have been augmented using inter-positional bone graft after ridge splitting and then evaluated clinically and radiographically using CBCT. A core Biopsy was harvested from all augmented sites in both groups for histological assessment. Patients attended the follow-up recalls and visits till the end of the study period. All patients were included for statistical analysis.

### Clinical findings

#### Primary surgical phase

In the primary surgical phase, no neurosensory deficits were recorded in the donor site except in one case in the intervention group, who suffered from partial paraesthesia immediately postoperative but recovered spontaneously a month later. In the conventional group, the splitting and packing of mixed bone particles inter-cortically were performed with no complications except in one case where the labial wall was separated unintentionally during the over-packing of the graft. However, the split cortex was still attached to the periosteum. Proper suturing and immobilization were carried out for appropriate healing.

### Secondary surgical phase

Six months postoperatively, all patients showed uneventful soft-tissue healing with no signs of infection. Upon surgical exposure to the augmented ridges, all cases revealed sufficient bone width that allowed conventional implant placement. However, small remnants of xenogeneic bone particles were still present, which did not participate in bone formation in the conventional group. One conventional case required additional bone augmentation using guided bone regeneration after implant placement in the same visit to cover the exposed implant threads.

### Radiographic findings

#### Preoperative versus immediate postoperative bone width (Gained bone Width)

In the control group, the mean value of the preoperative bone width was (6.18 ± 1.22) compared to (8.35 ± 1.72) immediately postoperatively. This bone width increase was statistically significant with (< 0.001) p-value. Also, the mean value of the preoperative bone width compared to the immediate postoperative bone width in the intervention group was (6.08 ± 0.75) and (7.52 ± 0.95) respectively, which also showed a statistically significant increase in immediate postoperative bone width with (< 0.001), P-value. So, both groups had a significant increase in bone width immediately postoperatively, but it was significantly higher in the control group (*p* < 0.001). Table 1.

**Table 1:** Intragroup comparisons and summary statistics for bone width (mm) (A)

Group	Gained bone width (Mean ± SD) (mm)		*p*-value
	Pre-operative	Immediately post-operative	
Control	6.18 ± 1.22	8.35 ± 1.72	< 0.001*
Intervention	6.08 ± 0.75	7.52 ± 0.95	< 0.001*

*; significant (*p* < 0.05) ns; non-significant (*p* > 0.05)

### Immediate versus 6 months postoperative bone width (Maintained bone Width)

The mean immediate postoperative bone width in the control group was (8.35 ± 1.72) and was reduced to (7.14 ± 1.65) 6 months postoperative; this reduction in bone width was statistically significant (*p* < 0.001). While the mean immediate postoperative bone width in the intervention group was (7.52 ± 0.95) and was reduced to (7.10 ± 1.00^A^) 6 months postoperative, this reduction in bone width was statistically non-significant (*p* = 0.135). So, the maintained bone width 6 months postoperative was significantly higher in the intervention group than in the control group. Table 2.

**Table 2:** Intragroup comparisons and summary statistics for bone width (mm) (B)

Group	Maintained bone width (Mean ± SD) (mm)		*p*-value
	Immediately post-operative	After 6 months	
Control	8.35 ± 1.72	7.14 ± 1.65	< 0.001*
Intervention	7.52 ± 0.95	7.10 ± 1.00^A^	**0.135ns**

*; significant (*p* < 0.05) ns; non-significant (*p* > 0.05)

### Preoperative versus final bone width

The mean preoperative bone width in the control group was (6.18 ± 1.22) and was increased to (7.14 ± 1.65) 6 months postoperative, which was not statistically significant (*p* = 0.211). The mean preoperative bone width in the intervention group was (6.08 ± 0.75) and was increased to (7.10 ± 1.00) 6 months postoperative, which was statistically significant (*p* < 0.001). So, the final bone width was higher and statistically significant in the intervention group than in the control group. Table 3.

**Table 3:** Intragroup comparisons and summary statistics for bone width (mm) (C)

Group	Bone width (Mean ± SD) (mm)		*p*-value
	Pre-operative	After 6 months	
Control	6.18 ± 1.22	7.14 ± 1.65	**0.211ns**
Intervention	6.08 ± 0.75	7.10 ± 1.00	< 0.001*

*; significant (*p* < 0.05) ns; non-significant (*p* > 0.05)

### Histological and histomorphometric findings

The microscopically examined stained sections revealed the high-quality mature bone with well-formed havarsian canals in the intervention group. Meanwhile, the conventional group showed prevalent osteoid bone with abundant remnants of xenograft (Fig. 7). The mean area of new bone (%) was (19.76 ± 3.90) for the control group and (50.32 ± 9.7) for the intervention group. So, the intervention group had a significantly higher value of newly formed bone (%) than the control group (*p* < 0.001). Table 4.

**Table 4:** Intergroup comparisons and summary statistics for area of new bone (%)

Area of new bone (Mean ± SD (%)		*p*-value
Control	Intervention	
19.76 ± 3.90	**50.32 ± 9.70**	< 0.001*

*; significant (*p* < 0.05) ns; non-significant (*p* > 0.05)

## Discussion

The absence of teeth combined with the loss of facial bone support in the anterior aesthetic zone significantly complicates the process of edentulous ridge rehabilitation since both aesthetics and function are severely impaired. Therefore, restoring missing teeth is crucial for enhancing the patients’ aesthetic, functional, and psychological well-being [13, 14]. Several surgical procedures have been described in the literature for horizontal ridge augmentation with proper implant placement in different resorption patterns of the horizontally deficient anterior maxilla, such as guided bone regeneration (GBR), only bone block grafting, ridge expansion, and ridge splitting. Each surgical technique has its advantages and limitations, which influence the surgeon’s preference based on aspects such as resorption pattern, bone type, ridge dimensions, and the patient’s medical and economic status [2, 15–37].

The current study was conducted to be a randomized controlled clinical trial that followed CONSORT guidelines. The recruited patients were selected based on the criterion that the Ridge should have a minimum of 3 mm bone width. The 3 mm measurement of bone consisted of a minimum of 1 mm trabecular bone located between the cortical plates. This ensures a minimum thickness of 1.5 mm of bone, consisting of both cortical and cancellous bone, on both sides of the divided cortices. This thickness is necessary to facilitate bone splitting and ensure a sufficient blood supply for proper healing. Additionally, the facial aspect of the labial cortical plate must be nearly straight without scooped-out labial wall defects to allow integral split cortical plates and limit the incidence of bad splitting [16].

The literature introduced many techniques for class IV Cawood and Howell augmentation [2, 15–37]. In the current study, ridge splitting was chosen over other augmentation techniques as in ridge splitting; unlike the onlay grafting technique and GBR, the harvested bone graft is placed between two walled vascularized bony beds, resulting in improved vascularization and reduced resorption of the sandwiched bone graft while the onlay bone graft is resting on a single wall of vascularized bony bed with subsequent limited blood supply for the graft resulting into further graft resorption [17]. Similarly, ridge splitting was preferred over the GBR technique to overcome space collapse caused by compressible resorbable membranes, which lack stability and rigidity [18, 19]. Moreover, ridge splitting avoids dehiscence, exposure, and infection, which are common complications that can arise with non-resorbable membranes or meshes [20].

Based on the literature, non-staged ridge splitting is a preferred technique in which the implant is placed simultaneously to serve as spacers and prevent the collapse of split cortices [21–23]. However, the aesthetic need for prosthetic-driven implant placement in the anterior maxilla elucidates a significant limitation in ridge splitting combined with simultaneous implant placement, as the exaggerated labial inclination of the placed implants adversely affects the aesthetic outcome of the prosthesis [24]. Necessitating the use of screw-retained restoration with the presence of an unaesthetic labial screw channel orifice or the use of cement-retained prostheses, which does not maintain healthy periodontal tissue around the implant [39]. Moreover, the labially inclined implants affect the labial wall, causing later labial bone resorption and subsequent implant failure [38, 39]. So, the current study employed a two-stage approach with delayed implant placement in an attempt to achieve enhanced bone quantity and quality, as well as improved aesthetic outcomes.

The main source of controversy in the staged ridge-splitting technique lies in the choice of grafting material source, form, and consistency [25–28]. In the current study, a mixture of graft materials was used in the control group in accordance with González-García et al., who used a mixture of autogenous mandibular symphyseal bone particles and bovine bone particles, which showed predictable results in terms of bone formation [29]. While in the study group, an autogenous mandibular symphyseal bone block in the form of a bone wedge was placed in between the split cortices with careful malleting to minimize the danger of the labial cortex fracture in accordance with Atef et al. and Pénzes et al., The hypothesis behind the use of intercortical bone wedge in the current study is to increase the graft-to-host contact area, enhancing graft-host interaction with subsequent graft bed incorporation. As well as the mechanical ability of the bone wedge to guard against the predicted collapse of the split cortices [11, 30]. Moreover, the harvested bone from the mandibular symphysis in the current study exhibits a corticocancellous nature. This particular type of bone facilitates accelerated vascularization, hence enabling enhanced growth of blood vessels within it. Consequently, when this bone wedge is wedged within the recipient’s bone bed, it incorporates more efficiently [31].

Immediate postoperatively, the increase in bone width in the control group was higher than in the intervention group. This increase is owing to the over-packing of the bone particles inter-cortically to compensate for the predicted cortical collapse and later bone resorption. In contrast, in the intervention group, the gained bone width was predetermined based on the thickness of the harvested bone wedge and the degree of wedge malleting, as the deeper the wedge is malleted, the more the divergence of the split cortices and more width will be obtained.

Both groups in the second stage had bone dimensions suitable for implant placement, according to M Alami, 2024 [32]. However, the control group experienced a significant reduction in the gained bone width 6 months postoperatively. This reduction can be attributed to the collapse that occurred due to inadequate mechanical support provided by the particulate bone graft. This is consistent with Gultekin BA et al. Who demonstrated the difference in resorption rates on grafting using bone blocks versus bone particles [33]. In contrast, the intervention group maintained the gained bone width with better graft incorporation and less resorption compared to the control group. This is consistent with Rahhal et al., who demonstrated the promising results of inter-positional bone blocks for horizontal ridge augmentation [6].

The higher maintained bone width in the intervention group is attributed to the mechanical support of the snugly fitted autogenous bone wedge, which protects the split cortices against the collapse that could result from the induced stresses during the healing period. Additionally, this mechanical support enhanced the graft stability and allowed for intimate contact between the graft and the surrounding vascularized graft bed. These results are consistent with the results of the study conducted by Atef et al., who compared onlay bone grafts versus inter-positional bone grafts placed between divided mandibular cortices. Also, Lustmann and Lewinstein reported a widening approach utilizing alveolar ridge splitting and inter-positional autogenous bone graft in the mandibular molar area [11, 34].

Regarding the histomorphometric examination, the intervention group had a higher area of newly formed mature bone than the control group (*p* < 0.001). This can be attributed to the improved blood supply to the snugly fitted autogenous bone wedge. This enhanced blood flow promotes the process of bone formation, and these findings are consistent with Rocchietta et al. [35]., Who demonstrated that block grafts outperform the particulate grafts histologically in the form of bone fill values.

Even though the wedge group had a higher value in maintaining the gained bone width after 6 months, the Prolonged treatment period is one of the technique’s drawbacks, in addition to the morbidity to the donor site with the limited amount of bone that could be harvested intraorally. Further studies using allogenic bone wedges are recommended to overcome those limitations.

## Conclusions

The two-stage Ridge splitting procedure using an interposition bone wedge is an effective method for horizontal ridge augmentation of defective alveolar ridges in the aesthetic zone. It has well-established results of bone width gain in addition to higher quality and quantity of the newly formed bone.

## Electronic supplementary material

Below is the link to the electronic supplementary material.

Supplementary Material 1

## Data Availability

All data are available whenever requested from the corresponding author of the manuscript. Email: (yasmin.ahmed@dentistry.cu.edu.eg)
